# Tumor specific delivery and therapy mediate by integrin β6-target immunoliposomes for β6-siRNA in colon carcinoma

**DOI:** 10.18632/oncotarget.13209

**Published:** 2016-11-08

**Authors:** Liu Song, Zhang Fan, Niu Jun, Liang Benjia, Li Zequn, Wang Xilong, Jia Zhongming, Han yong, Wang Xiaohong, Cheng Kai, Yang Zhenlin

**Affiliations:** ^1^ Department of Thyroid & Breast Surgery, Binzhou Medical College Affiliated Hospital, Binzhou 256600, Shandong, PR China; ^2^ Department of Oncology, Binzhou Medical College Affiliated Hospital, Binzhou 256600, Shandong, PR China; ^3^ Department of Hepatobiliary Surgery, Qilu Hospital of Shandong University, Jinan 250012, Shandong, PR China

**Keywords:** colon carcinoma, integrinβ6, target therapy, immunoliposomes, siRNA

## Abstract

Adjuvant chemotherapy does not achieve the desired therapeutic efficacy in colon cancer as a result of the deficient reaction. Gene therapy using small interfering RNAs (siRNAs) delivered by target delivering system represents a potent and specific strategy in tumor therapy. Integrinβ6 is exclusively expressed in malignant colonic epithelia, associated with the progression, metastasis, and chemotherapeutic resistance of colon cancer. Accordingly, designing an efficient and targeted delivery system for β6-siRNA could be a potential approach to improve therapeutic efficacy of colon cancer. Here, we designed the Integrinβ6 target immunoliposomes for highly efficient and selective delivery of β6-siRNA in colon cancer, which consequently resulted in greatly growth suppression, invasion and metastasis of colon cancer cells. Moreover, it was able to greatly inhibit the tumor growing in vivo.

## INTRODUCTION

Colon cancer is the third most common cancer and the fourth leading cause of cancer-related deaths worldwide. Each year, more than 1.2 million people are diagnosed with colon cancer, and even more than 600,000 die from the disease [1]. Although surgery remains the preferred treatment, adjuvant chemotherapy is the conventional care for patients with metastasis to palliate symptoms and reduce mortality [2, 3]. However, because of the subsequent chemo-resistance, the five-year survival rate of these cases remains low. Therefore, it is urgent to explore alternative and effective strategies for colon cancer treatment.

Gene therapy using small interfering RNAs (siRNAs) represents a potent and specific strategy in tumor therapy. The siRNAs are 21-25 nucleotide (nt), double-stranded RNAs and finding increasing applications due to their role of transcript degradation for silencing tumor-specific genes and easy design strategies [4]. However, as a negative-charged and water-soluble macro molecule, application of free siRNAs faces many barriers such as ribonuclease (RNase) degradation, poor permeability and sub-optimal bioavailability, which impede the clinical success of siRNAs therapeutics [5]. Among the existing arsenal of siRNAs delivery reagents, lipid-based delivery systems, such as liposomes, prove to be effective vectors of choice for systemic siRNAs delivery in tumor therapy [6]. Moreover, the short circulation lifetime of liposomes could be overcome by attaching polymeric PEG on the surface [7, 8].

Nevertheless, PEGylation represents a major disadvantage for nucleic acid internalization and endosomal escape, which leads to greatly reduced transfection efficiency. In addition, these liposomes passively interact with targeted cells, in vitro or in vivo, resulting in nonspecific siRNAs release [9]. Therefore, to further improve the transfection efficiency of PEGylated liposomes, it is a practical way to develop immunoliposomes conjugated with targeting ligands [10, 11].

Integrinβ6 is a subtype of integrin that is expressed exclusively on the surfaces of epithelial cells and is a receptor for extracellular matrix proteins. Integrinβ6 expression is upregulated during embryogenesis, oncogenesis and epithelial repair, whereas it is generally undetectable in healthy epithelial tissues [12, 13]. In colon cancer, integrinβ6 is specifically expressed in tumor tissues and is rarely present in tissues adjacent to the tumor. In addition, integrinβ6 is associated with colon cancer pathology, malignancy, and TNM stage and could act as a prognostic indicator in aggressive colon carcinomas [14, 15]. Our research previously confirmed that integrinβ6 contributed to chemotherapeutic resistance in colon cancer. Unsurprisingly, the exclusive expression of integrin β6 and its influential effects in colon cancer make it a novel therapeutic target for colon cancer treatment [16, 17].

We have previously developed integrinβ6-targeted immunoliposomes (TLPs) which could provide a highly efficient approach for targeted drug delivery and enhance the antitumor efficiency in colon cancer [18]. However, research concerning integrinβ6-targeted immunoliposomes used for siRNAs delivery has not been reported. In the current study, we use integrinβ6-targeted immunoliposomes as a siRNAs delivery system, and explore their transfection efficiency in colon cancer cells. Since we have previously confirmed that suppression of integrinβ6 by siRNAs inhibited cell growth and invasion in colon cancer cells, in this study, we additionally study the effects of integrinβ6-targeted immunoliposomes on cell death and migration/invasionin colon cancer when delivering the β6-siRNA.

## RESULTS

### Preparation and characterization of β6-targeted immunoliposomes

**Figure 1:**
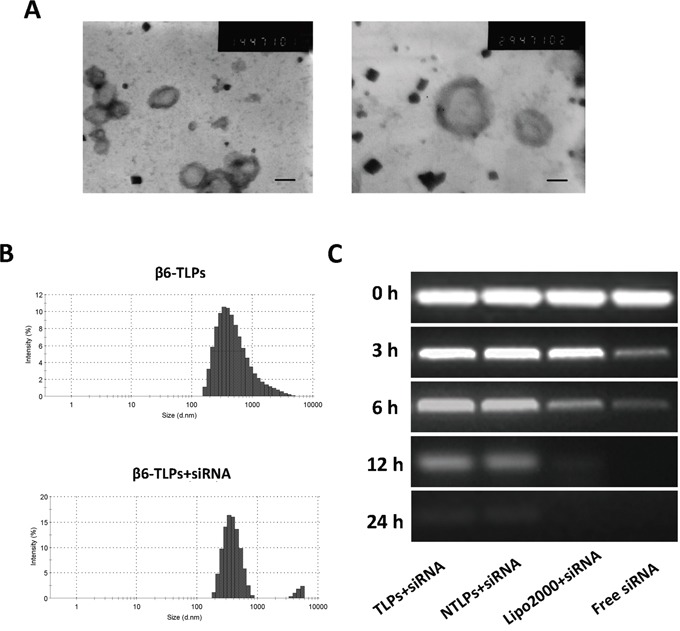
**A.** Transmission electron photomicrograms of β6-siRNA loaded TLPs. Bar = 200nm and 100nm. **B.** Typical particle size and distribution of β6-TLPs and β6-TLPs loaded with siRNA. **C.** siRNA serum stability assay. Samples of siRNA in aqueous solution or in liposomes were mixed with fresh serum at a 1:1 volume ratio. Each sample containing 0.25 μg siRNA were added onto a gel and electrophoresis was performed to visualize intact siRNA at different fixed times.

Integrinβ6-TLPs were successfully synthetized in our study as previously described [18]. The mean particle size of the TLPs was 385.3 ± 4.85 nm. The zeta potential was -7.65 ± 1.12 mV. After siRNA was loaded, β6-TLPs were still spherical or ellipsoidal, as shown directly by TEM (Figure 1A). Size distribution of β6-siRNA loaded TLPs was shown in Figure 1B, and the mean particle size was 396.1 ± 2.16 nm, which was slightly greater than that of free TLPs. These indicated that the load of siRNA has no significant impact on immunoliposomes size which played great role in circulation time and cellular uptake efficiency. The zeta potential of the siRNA loaded TLPs was also investigated. When loading with siRNA, the zeta potential of TLPs decreased to a value of -14.18 ± 1.74 mV.

In addition, the ultra-filtrating method was used to calculate the siRNA encapsulation efficiencies (EE%) of both non-target liposomes (NTLPs) and TLPs. The siRNA EE% of both liposomes was were 85.43% ± 2.03% and 87.54% ± 1.32%, respectively, suggesting that antibody conjugation have little adverse impact on siRNA EE%.

Furthermore, we detected the siRNA serum stability in order to confirm whether siRNA in liposomes were stable to digestion by nuclease in serum for transfection activity in cells [19]. In this study, we tested the stability of siRNA in Lipo2000, NTLPs and TLPs formulations by incubating siRNA loaded liposomes with an equal volume of serum and incubated at 37°C. As shown in Figure 1C, free siRNA started to degrade after 3 h and was completely degraded after 6 h. However, siRNA in Lipo2000 started to degrade after 6 h and fully degraded even after 12 h. In contrast, siRNA fully degradation in both NTLPs and TLPs happened after 24 h, which indicated that NTLPs and TLPs equally protected siRNA from serum degradation to a great extent.

### In vitro cellular uptake and transfection efficiency of β6-targeted immunoliposomes

**Figure 2:**
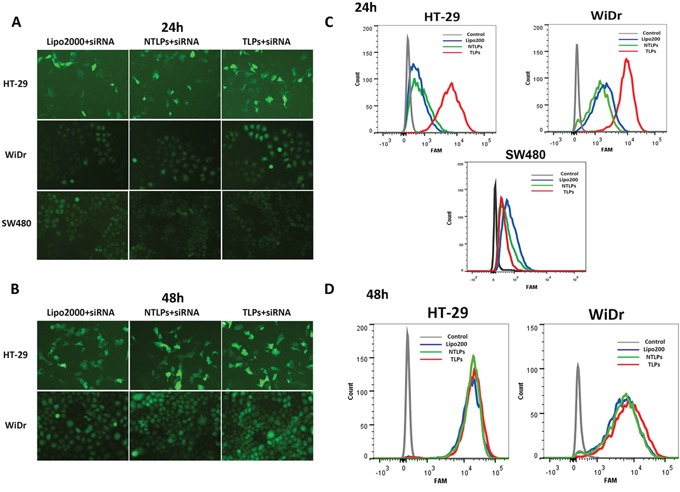
Uptake of siRNA liposomal complexes in colon cancer cells **A** and **B.** Fluorescent micrographs of HT-29, WiDr and SW480 cells transfected with FAM-siRNA loaded Lipo2000, NLPs and TLPs with the final siRNA concentration of 100 nM at 24 and 48 hours post transfection. **C** and **D.** Cellular uptake of siRNA were also evaluated by flowcytometry.

In order to analyze the transfection efficiency of β6-targeted immunoliposomes, we examined the in vitro cellular uptake of siRNA encapsulated in liposomes. Colon cancer cells HT-29, WiDr and SW480 were treated with FAM-siRNA loaded Lipo2000, NTLPs and TLPs for 24 h and 48 h. Under a fluorescence microscope, the green fluorescence of the FAM-labeled siRNA was visualized. As shown in Figure 2A and 2B, siRNA in the liposomes were all successfully taken up by HT-29 and WiDr cells, and obviously increased from 24h to 48h. The siRNA in TLPs had a higher cellular uptake than both Lipo2000 and NTLPs in 24 hours. However, there was no significant difference among these three liposomes formulations at 48 hours after treatment. However, when treated on SW480 cells which had no integrin β6 expression, TLPs has a similar cellular uptake of siRNA with NTLPs and Lipo2000 in 24 h.

Flow cytometry was used to further quantify the cellular uptake amount of siRNA (Figure 2C and 2D). Slight fluorescence was detected in HT-29 and WiDr cells treated with FAM-siRNA encapsulated in Lipo2000 and NTLPs at 24 h point, whereas much higher fluorescence intensities were measured in both cell lines treated with TLPs loaded with FAM-siRNA. However, there was no significant different among TLPs, NTLPS and Lipo2000 on SW480 cells. As time went to 48 hours, the difference began to narrow in these three groups. All above results indicated that TLPs might be able to promote the cellular uptake of siRNA in β6-expressing colon cancer cells, which probably resulted in improved β6 gene silencing. Moreover, the decreased cellular uptake of TLPs on SW480 cells that expressed negative β6 may testify the specific cell binding of TLPs.

### Integrinβ6 gene silencing in colon cancer cells

**Figure 3:**
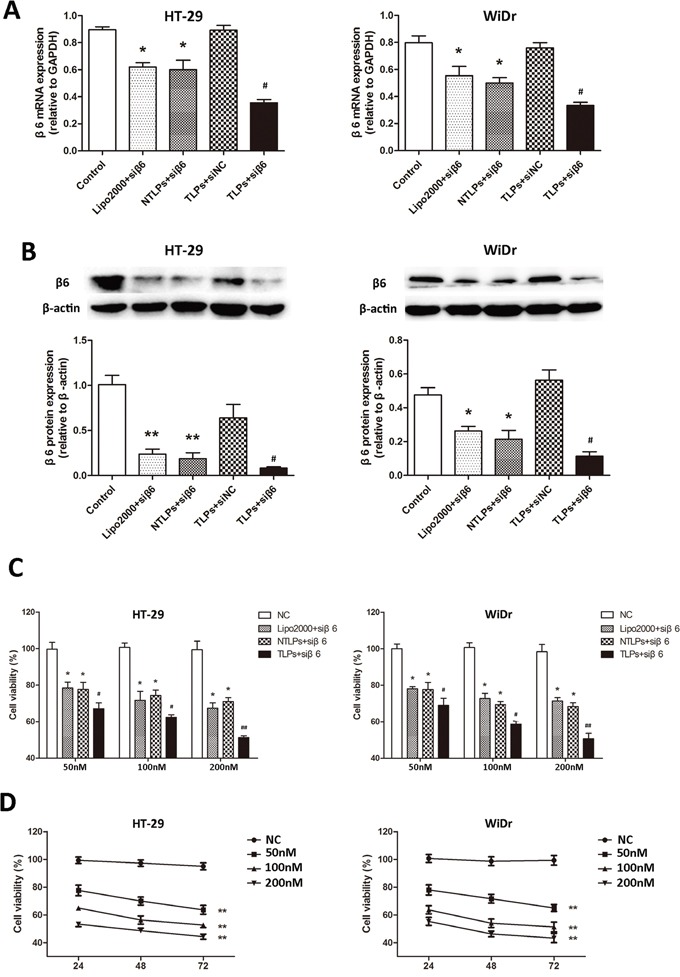
**A.** The mRNA expression of integrinβ6 in HT-29 and WiDr colon cancer cell lines was examined by quantitative realtime PCR. β6-siRNA transfected with TLPs had a greater suppression of β6 expression in both cell lines. **B.** Western blot analysis of integrinβ6 protein expression. The relative protein levels were expressed as the ratio of β-actin. The data are expressed as the means ± SD of three independent experiments, * P <0.05, ** P < 0.01 compared with control, ^#^ P < 0.05 compared with NTLPs. **C.** Growth inhibition in cells was evaluated by the CCK-8 assay. HT-29 and WiDr cells were treated with negative agents (negative control, NC), β6-siRNA loaded Lipo2000, NTLPs and TLPs at different concentrations (50, 100 and 200 nM). At 48 hours after treatment, the CCK-8 assay was performed. **D.** Both cells were transfected with serial concentrations of β6-siRNA-TLPs (50, 100 and 100 nM), and cell viability was assessed at 24, 48 and 72hours. The data are expressed as the means ± SD of three independent experiments, * P < 0.05, ** P < 0.01 compared with control, ^#^ P < 0.05, ^# #^ P <0.01 compared with NTLPs.

We have reported that integrinβ6 played a significant role in cell growth, invasion, and metastasis of colon cancer which were able to be inhibited by suppression of integrinβ6 expression [20-22]. Therefore, finding an effective and integrinβ6 targeted method to suppress the β6 expression might be provide a potential way for therapy of colon cancer. In order to examine the efficiency of β6 gene silencing of TLPs, we detected the β6 expression in HT-29 and WiDr cell lines treated with β6-siRNA encapsulated in Lipo2000, NTLPs and TLPs in RNA level by PCR. As shown in Figure 3A, all the siRNA liposomal complex could decrease the expression of β6. However, comparing with Lipo2000 and NTLPs, the β6 mRNA expression in both cells lines was greatly reduced in TLPs group which suggested that TLPs could promote the expression suppression of β6-siRNA. However, there was no obvious difference between Lipo2000 and NTLPs, which indicated that PEGylated liposomes were not able to increase the transfection efficiency of siRNA in vitro.

In addition, we examined the integrinβ6 protein expression by western blotting. HT-29 and WiDr were treated as described above. It was also notable to see in Figure 3B that β6-siRNA in TLPs was more likely to inhibit the β6 expression than both Lipo2000 and NTLPs. In consistence with the result of PCR, Lipo2000 and NTLPs had an equal activation of β6 expression suppression. The β6 scrambled siRNA in TLPs had no effects on the expression of integrinβ6.

### Cell viability

Our previous study showed that β6-siRNA was able to suppress the growth of colon cancer cells. To explore whether β6-TLPs could improve the effect on tumor suppression of β6-siRNA, HT-29 and WiDr cells were exposed to β6-siRNA in Lipo2000, NTLPs and TLPs three liposomal formulations, with β6-siRNA at different concentrations (50nM, 100nM and 200 nM). The β6 scrambled siRNA in TLPs were used as controls. The cytotoxicity and growth inhibition of the cells was then evaluated by the CCK-8 assay. As shown in Figure 3C, the cell growth was notably suppressed by the β6-siRNA loaded in all three liposomal formulations at each dose for 48 hours. However, TLPs had the lowest cell viability (P < 0.05), while the NTLPs did not induce a marked growth suppression effect compared with the Lipo2000.

Moreover, the TLPs demonstrated a clear β6-siRNA dose- and time-dependent cytotoxicity in the experimental cell lines (Figure 3D). All these results suggested that the β6-TLPs enhanced the cell growth suppression of β6-siRNA in colon cancer cells, which probably resulted from the more effective delivery and cellular uptake of TLPs.

### β6-siRNA TLPs induce apoptosis of colon cancer cells

**Figure 4:**
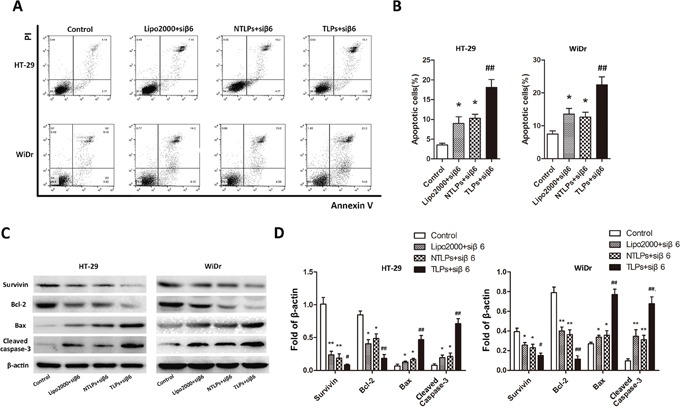
Efficacy of β6-siRNA-TLPs on the apoptosis of colon cancer cells **A.** HT-29 and WiDr cells were treated with negative agents (negative control, NC), β6-siRNA loaded Lipo2000, NTLPs and TLPs. Cell apoptosis was detected by Annexin V-FITC/PI double-staining assay with flow cytometry. **B.** Quantification of apoptosis were also showed. **C.** Western blot assays showed that β6-siRNA loaded in TLPs led to a distinct up-regulation of Bax and cleaved caspase-3 and down-regulation of Bcl-2, pro-caspase-3 and Survivin. **D.** Quantification of expression were also showed. The data are expressed as the means ± SD of three independent experiments, * P < 0.05, ** P < 0.01 compared with control, ^#^ P < 0.05, ^# #^ P <compared with NTLPs.

The integrinβ6 could promote the cell growth and protect cell apoptosis of colon cancer [16]. Therefore, to investigate the effect of the β6-siRNA which delivered by β6-TLPs on cell apoptosis, HT-29 and WiDr cells were transfected Lipo2000, NTLPs and TLPs entrapping β6-siRNA with the final siRNA concentration 100nM. Subsequently, the cellular apoptotic rate was analyzed through Annexin V-FITC and PI double staining using flow cytometry. The apoptotic rates induced by β6-TLPs (HT-29 17.74%; WiDr 24.64%) were notably increased in comparison with those of Lipo2000 and NTLPs (P < 0.05; Figure 4A and 4B). However, NTLPs presented similar β6-siRNA induced cell apoptosis with Lipo2000. These findings indicated that the TLPs could improve the cell apoptosis due to the promoted transfection of β6-siRNA.

To further explore the potential molecular mechanisms by which the β6-siRNA loaded β6-TLPs induce cell apoptosis, HT-29 and WiDr cells were grouped and treated as described in the section above, and apoptosis-related proteins were detected by Western blotting (Figure 4C and 4D). The results showed that the expression of cleaved caspase-3 and Bax were all increased in the treatment groups in both HT-29 and WiDr cells. However, TLPs group had a higher level of caspase-3 and Bax expression than Lipo2000 and NTLPs. Additionally, the protein levels of Bcl-2 and Survivin were reduced in all the mentioned groups, while β6-siRNA in TLPs was more likely to lower the expression. However, there were no significant differences between the Lipo2000 and NTLPs groups.

### β6-siRNA TLPs inhibit colon cancer cell migration and invasion

**Figure 5:**
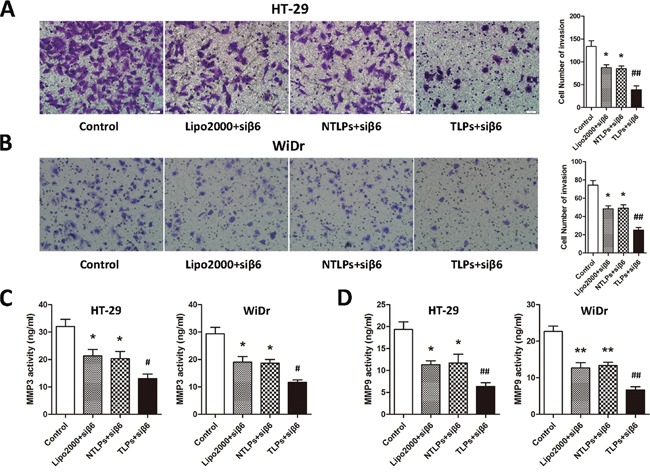
**A and B.** Effects on tumor cell migration and invasion ability were analyzed with Transwell assay. In comparison with Lipo2000 and NTLPs, TLPs was able to improve the β6-siRNA suppression on cell migration and invasion of both HT-29 and WiDr cell lines. Meanwhile, TLPs loaded with β6-siRNA could decrease the activity of MMP3 and MMP9 in HT-29 and WiDr cell lines. **C** and **D.** * P < 0.05, ** P < 0.01 compared with control, ^#^ P < 0.05, ^# #^ P <0.01 compared with NTLPs.

We have previously found that integrinβ6 was able to induce the migration and invasion of colon cancer cells by up-regulation of MMP-3/MMP-9 [23, 24]. In the meantime, the migration and invasion of colon cancer cells could be suppressed by β6-siRNA. In this study, in order to determine whether β6-TLPs could promote the suppression efficiency of β6-siRNA on migration and invasion of colon cancer cells, transwell assay was used to examine the migration and invasion with the membrane without (migration) Matrigel or pre-coated with (invasion). As shown in Figure 5A, β6-siRNA delivered in TLPs greatly decreased the migration of both HT-29 and WiDr cell lines in comparison with Lipo2000 and NTLPs (P < 0.01). Meanwhile, the invasive ability of HT-29 and WiDr cells was dramatically inhibited in TLPs group (Figure 5B) (P < 0.01). Although β6-siRNA in Lipo2000 and NTLPs were both able to suppress the migration and invasion of these two cell lines, there was no obvious differences between these liposomal formulations. Moreover, we also detected the MMP-3 and MMP-9 expression level by ELISA. As shown in Figure 5C and 5D, β6-siRNA encapsulated in TLPs was more likely to inhibit the activation of MMP-3 and MMP-9 than Lipo2000 and NTLPs (P < 0.01), which possibly resulted in the higher suppression of migration and invasion of colon cancer cells.

### Tumor growth inhibition of β6-siRNA TLPs in vivo

**Figure 6:**
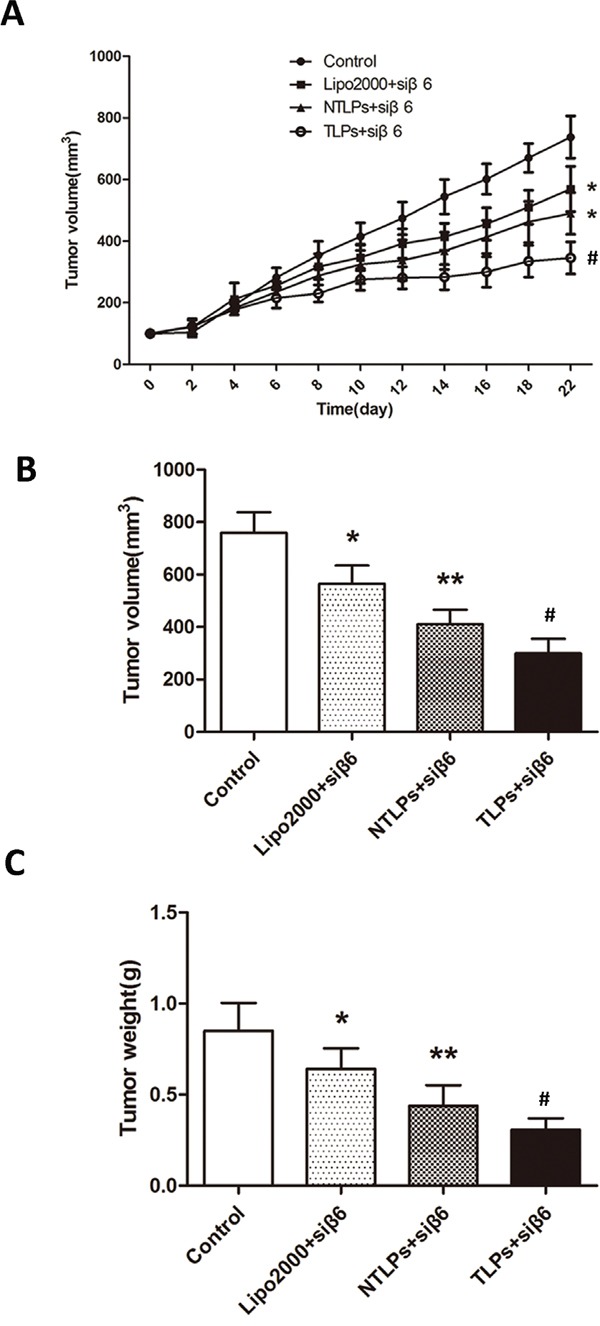
In vivo anti-tumor study of liposomes in HT-29 tumor-bearing nude mice after intratumor injection with β6-siRNA loaded Lipo2000, NTLPs and TLPs. The dosage β6-siRNA was 0.4 mg/kg **A.** Relative tumor volume time curve (n=5), *P < 0.05 compared with control, ^#^ P < 0.05 compared with NTLPs. **B** and **C.** After 3 weeks, the mice were sacrificed and the tumors were dislodged. Tumors were also measured and weighed. (n=5), * P < 0.05, ** P < 0.01 compared with control, ^#^ P < 0.05 compared with NTLPs.

To evaluate the siRNA anti-tumor efficacy of β6-TLPs in vivo, β6-siRNA loaded in Lipo2000, NTLPs and TLPs at the dose of siRNA 0.4 mg/kg was intratumorly injected in mice bearing HT-29 human colon cancer cell xenografts. The antitumor effect, indicated by tumor growth, was shown in Figure 6A and 6B. All treatments obviously suppressed tumor growth during the treatment period (P < 0.05). No antitumor effects were observed in the N.S. Moreover, the tumor suppression of β6-TLPs was significantly stronger than Lipo2000 and NTLPs (P < 0.05), while the NTLPs had a higher tumor suppression than Lipo2000 (P < 0.05). Consistent with the above result, the average tumor weight in mice treated with β6-siRNA loaded in TLPs was approximately 2-fold lower than that in mice treated with β6-siRNA loaded in NTLPs (P < 0.05; Figure 6C).

**Figure 7:**
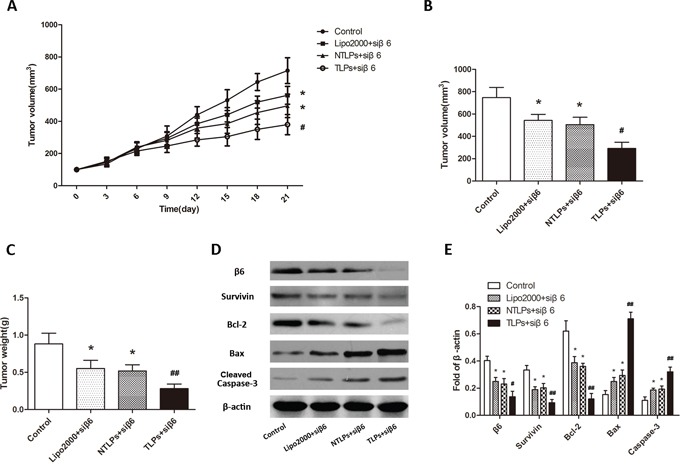
Anti-tumor efficiency of liposomes in HT-29 tumor-bearing nude mice after intravenous administration with β6-siRNA loaded Lipo2000, NTLPs and TLPs **A.** Relative tumor volume time curve (n=5), *P < 0.05 compared with control, ^#^ P < 0.05 compared with NTLPs. **B** and **C.** Tumors were also measured and weighed. (n=5), *P < 0.05 compared with control,^#^ P < 0.05, ^##^ P < 0.05 compared with NTLPs. **D** and **E.** Western blot assays detected the expression of integrinβ6 and apoptosis related proteins, such as Survivin, Bcl-2, Bax and cleaved caspase-3 in tumor tissues. (D) Quantification of expression were also showed. The data are expressed as the means ± SD of three independent experiments, * P < 0.05, ** P < 0.01 compared with control, ^#^ P < 0.05, ^# #^ P <compared with NTLPs.

Moreover, in order to furtherly testify the tumor-specificity of the β6-TLPs, β6-siRNA in three different liposomes was intravenously injected in the same HT-29 human colon cancer model. As shown in Figure 7A and 7B, the tumor growth is significantly suppressed in β6-TLPs group in comparison with Lipo2000 and NTLPs, which was consistent with the result of β6-TLPs intratumorly injection (P < 0.05). However, there was no obvious difference between Lipo2000 and NTLPs groups. These results may indicate that the improved anti-tumor efficiency of TLPs could contribute to the specific binding and higher delivery efficiency of TLPs. Meanwhile, β6 expression was greatly reduced in TLPs groups (Figure 7D and 7E). The expression of apoptosis related proteins of the tumor tissues was also detected in order to explore the potential mechanisms. As shown in Figure 7D and 7E, TLPs group had a higher level of expression of cleaved caspase-3 and Bax and lower level of Bcl-2 and Survivin.

## DISCUSSION

Like all the other targeting antigens having been already widely exploited, integrinβ6 also holds the basic characteristic as a target for target therapy in tumors that is abundant expression in solid tumors and limited expression in normal epithelial tissues [14]. Beyond that, integrinβ6 has its own special tumor-related properties which make it an ideal targeting receptor for tumor therapy, especially for colon cancer. We have previously reported that integrinβ6 was associated with the malignancy of colon cancer and it would act as an indicator for inclination of metastasis and poor prognosis [15]. Integrinβ6 remained high level at the invasive edges between aggressive tumor site and surrounding tissues, and also up-regulated its own expression when cancer cells becoming dense [25]. Moreover, integrinβ6 had the endocytosis and recycling procedure which promote colon cancer cell migration [26]. Therefore, there is no doubt that integrinβ6 emerges as an attractive new candidate as a therapeutic target which will definitely improve therapeutic efficacy on colon carcinoma.

Based on the important role the integrin β6 played in colon cancer, in the current study we developed the β6-targeted immunoliposomes loaded with β6-siRNA which could not only increase the cellular internalization of siRNA in β6-positive colon cancer cells, but also suppress the cancer by silencing the integrinβ6 expression. As what we found in this study, TLPs which targeted integrin β6 was able to increase the cellular internalization of siRNA on β6 expressing colon cancer cells. And the liposome-cell binding was integrin β6 specific because the cellular uptake of siRNA would vanish if there was no β6 expression on the cell surface (Figure 2A and 2B). Additionally, TLPs only promoted the siRNA internalization at the early stage (24h) and all the three liposomes finally had the equal volume of siRNA in the cells at 48h. This might suggest that TLPs increased the efficiency of the siRNA cellular internalization instead of the total number of the siRNA. Then the expression of the integrin β6 in both mRNA and protein level were detected to confirm whether the improved cellular internalization of TLPs could lead to the down-regulation of β6 expression. Obviously, β6-siRNA delivered in TLPs was more likely to enhance the efficiency of β6 gene silencing in colon cancer cells compared with non-target liposomal formulations (Figure 3A and 3B).

In our previous studies, down regulation of integrinβ6 expression by using β6-siRNA could suppress the cell viability and promote apoptosis of colon cancer cells. Accordingly, we speculate that the TLPs which enhanced the β6-siRNA cellular uptake and β6 gene silencing could possibly result in more obvious inhibition of colon cancer cell progression. As shown in Figure 3C and 3D, TLPs was proved to raise up the suppression of cell viability in comparison with NTLPs and lipo2000. In the same time, TLPs also accelerated the apoptosis of colon cancer cells (Figure 4A and 4B). Integrinβ6 was reported to inhibit apoptosis of colon cancer cells through regulating the expression of apoptosis associated proteins [16]. In this study, we found significantly elevated expression of Bax and cleaved Caspase-3 but reduced expression of Bcl-2 and Survivin in the β6 siRNA-TLPs group (Figure 4C). These data suggest that the TLPs accelerate the β6-siRNA induced apoptosis of colon cancer cells by increase the expression of apoptosis promoting proteins and decrease anti-apoptosis proteins in process of accelerating apoptosis of colon cancer cells.

Matrix metalloproteinases (MMPs) played fundamental roles in pathological processes through degradation of basal membranes and extracellular matrix, and some MMPs contributed to tumor invasion and metastasis [27, 28]. We have validated that integrinβ6 promoted the invasion, metastasis and degradation of extracellular matrix of colorectal cancer, thyroid carcinoma, gastric carcinoma, and pancreatic carcinoma through up-regulation of MMP-3/MMP-9 [29-31]. In this study, we explored the effects of β6-TLPs on the inhibition of colon cancer cell invasion and metastasis when delivering β6-siRNA. The β6-TLPs was able to prompt the effect of β6-siRNA on suppression the invasion and metastasis of HT-29 and WiDr colon cancer cells. Meanwhile, β6 siRNA-TLPs more significantly decreased the MMP-3/MMP-9 activity. These data suggested that β6-siRNA delivered in β6-TLPs was more likely to prevent the invasion and metastasis of colon cancer through inhibition of MMP-3/MMP-9 activity.

These promising in vitro results were further evaluated in vivo. The antitumor efficacy of β6-TLPs was significantly superior to that of both NTLPs and Lipo2000 when delivering β6-siRNA, which was probably caused by increased cellular internalization of β6-TLPs in tumor issues. The nontargeted liposomes like NTLPs and Lipo2000 passively interact with the tumor cells, eventually resulting in the delivery of the majority of siRNA into some normal tissues or cells rather than the tumor [32]. Moreover, TLPs still had a higher suppressive of the tumor growth when intravenous injection, which suggested that the TLPs could increase the β6-siRNA accumulation in tumor issues. In conclusion, the TPLs inhibit the tumor in vivo not only by increasing the β6-siRNA cellular uptake on tumor cells, but also enhancing the β6-siRNA accumulation in tumor issues.

In summary, this study successfully described a targeted delivery system for highly efficient and selective delivery of siRNA in colon cancer overexpressing integrinβ6 and protect the integrity of the siRNA from degradation and increase the cellular uptake efficiency in vitro. Consequently, the β6-TLPs resulted in greatly growth suppression and induction of apoptotic, when delivering β6-siRNA to integrinβ6 overexpressing colon cancer cells. We also observed an inhibited cell invasion of this system. Moreover, it could obviously suppress the tumor growing in vivo. Taken together, the β6-siRNA TLPs system may represent a potential strategy for the treatment of colon cancer.

## MATERIALS AND METHODS

### Materials

Hydrogenated soy phosphatidylcholine (HSPC) and cholesterol were purchased from Nippon Fine Chemical. Distearylphosphatidylethanolamine (DSPE)-mPEG (2000) and DSPE-PEG (2000)-NH_2_ were purchased from Avanti Polar Lipids. The homobifunctional crosslinker bis (sulfosuccinimidyl) suberate (BS3) was from ProteoChem. The monoclonal antibody that detects the extracellular domain of human integrin β6 (E7P6) was prepared and provided as a gift from Michael Agrez (The University of Newcastle, Newcastle, Australia). The cell counting kit-8 (CCK-8) was from Dojindo Molecular Technologies. The Annexin V–FITC/propidium iodide (PI) cell apoptosis apoptosisassay kit was purchased from Merck Millipore.

Small interference RNA (sense strand: 5‘-UUCUCC GAACGUGUCACGUTT-3’; antisense strand: 5‘-ACGU GACACGUUCGGAGAATT-3’) targeted to the human integrin β6 mRNA was synthesized and purified as described before with HPLC by GenePharma Co. Ltd (Shanghai, China) [17]. FAM-labeled negative control (NC) siRNA (FAM-siRNA) and scramble siRNA were also obtained from GenePharma (Shanghai, China).

### Cell lines

Human colon cancer cell lines HT-29, WiDr and SW480, were obtained from the ATCC. HT-29 and WiDr cells constantly express integrin β6. SW480 cells lack constitutive β6 expression. The cells were maintained as monolayers in medium comprising DMEM (Hyclone) containing10% heat inactivated FCS (Gibco) and supplemented with 20mmol/L HEPES, 100 IU/mL penicillin, and 100 mg/mL streptomycin. The cells were incubated in 37°C, 5% CO2, and saturated humidity.

### Preparation of siRNA loaded liposomes

The integrinβ6 targeted liposomes (TLPs) were prepared as reportedin our previous study with slight modifications. Briefly, HSPC, cholesterol, DSPE-PEG2000 and /DSPE-PEG2000-NH_2_ at a molar ratio of 2:1:0.08:0.02 were dissolved in a mixture of chloroform and methanol (9:1 v/v). Then the mixture in a ratio 4:1 (v/v) between organic and aqueous phase was sonicated at room temperature, and the chloroform and methanol were evaporated using a rotary evaporator. The lipid was hydrated with phosphate buffer saline (PBS, pH=7.4). The liposomes were shaken in a vortex to form an aqueous suspension and were subsequently extruded 10 times through polycarbonate filters with a defined pore size of 400–100 nm. Then integrinβ6-specific antibody E7P6 was coupled to the liposomes. The cross-linker BS3 was added to the liposomes at a final concentration of 5 mmol/L and was incubated for 1 hour at room temperature to activate the liposomes. The excess unreacted cross-linker was removed via ultrafiltration using an Amicon Ultra-0.5 3kD (Millipore). The activated liposomes were collected and incubated with integrinβ6 antibody (E7P6) for 2 hours at room temperature. Finally, 50 mmol/L Tris was added at room temperature for 15 minutes to quench the reaction. The immunoliposomes were separated from the unconjugated antibody using Sepharose CL-4Bcolumns.

Non-targeted liposomes (NTLP) composed of HSPC/Chol/DSPE-PEG2000 at a molar ratio of 2:1:0.1 were prepared with the same method as described above.

The siRNA was dissolved in DEPC-treated water at a final concentration of 20 μM. The liposomes and siRNA solution were mixed under gentle vortexing for 20 seconds and incubated for 20 minutes at room temperature for 30 min to ensure siRNA loading efficiently. The entrapment procedure was performed immediately before use.

### Characterization of liposomes

The transmission electron microscopy (TEM; JEM-100CX II, Japan) was used to observe the morphologies of TLP and NTLP. The mean particle size and zeta-potential values were analyzed by Malvern Zetasizer Nano ZS90.

### siRNA encapsulation efficiency (EE)

The EE of siRNA was determined by ultra-filtrating the FAM-siRNA loaded liposomes using Amicon Ultra-4 centrifugal filter devices (Millipore). After completely ultrafiltration, unencapsulated FAM-siRNA was collected and quantified by using a siRNA calibration line obtained with standard FAM-siRNA solutions. The fluorescence of FAM-siRNA was determined by the spectrofluorometer (Synergy^TM^4, USA) with the excitation and emission wavelengths of 495 and 525 nm. The siRNA EE was calculated from the equation: (M_i_-M_U_)/M_i_×100%. M_U_ and M_i_ were defined as the unencapsulated siRNA and totally added siRNA, respectively.

### Serum stability of liposomal siRNA

Serum stability of siRNA in liposomes versus in aqueous solution was determined using agarose gel electrophoresis as described before. Samples of siRNA in aqueous solution or in liposomes were mixed with fresh serum at a 1:1 volume ratio to obtain 50% serum concentration and incubated at 37°C. At different fixed times, aliquots containing 0.25 μg siRNA of each sample were added onto a gel and electrophoresis was performed to visualize intact siRNA.

### In vitro transfection efficiency

The cellular transfection efficiency was examined using a fluorescence microscope. Cells were seeded in 6-well plates. When cells density was up to approximately 50–60%, the medium was replaced with Opti-MEM (Gibco, USA) containing liposomal FAM-siRNA (the final siRNA concentration was 100 nM). The culture medium was replaced by complete medium after 6 hours of incubation. After 24 or 48 hours of treatment, the cellular fluorescence was visualized using a fluorescence microscope (Olympus, Japan). The cells were cultured and treated as described above. After 24 or 48 hours, cells were trypsinized and washed three times with PBS. Then, the cells were collected via centrifugation and were resuspended in 500 μl of PBS. Cellular uptake was analyzed by flow cytometry.

### Quantitative real-time PCR

Total RNA was extracted from cells by Trizol (Invitrogen). The reverse transcription reaction was performed using RevertAid™ First Strand cDNA Synthesis Kit (Fermentas), according to the manufacturer's instructions. cDNA obtained from reverse transcription reaction was analyzed by a real-time PCR thermocycler (IQ5 Real-Time PCR cycler; Bio-Rad Laboratories, Hercules, CA, USA) with SsoFast EvaGreen Supermix (Bio-Rad Laboratories). Quantitative values were obtained by the threshold cycle (CT) value. Relative mean fold change in expression ratios was calculated by the 2^-ΔΔCT^ method. Sequences of primers were as follows: integrin β6 (141 bp) forward primer, 5’- AGGATAG TTCT GTTTCCTGC -3’, β6 reverse primer 5’- ATCATAGGAATATTTGGAGG -3’. β-actin was used as an internal control.

### Western blotting

Cells were harvested and lysed. Protein samples at a final amount of 20 μg were loaded onto an SDS-PAGE gel and electrophoresed. Subsequently, the separated proteins were transferred to nitrocellulose membranes. The membranes were immunoblotted with primary antibody overnight at 4°C followed by HRP(horseradish peroxidase)-labeled secondary antibody. Immunoreactive bands were visualized using ECL method, and the optical density was analyzed with the Image Lab software. Values were expressed as a fold of β-actin.

### Cell viability assay

Cells were seeded in 96-well plates and transfected with TLPs, NTLPs or Lipo2000 entrapping integrinβ6-siRNA at a series of concentrations (the final siRNA concentration was 50nM, 100nM and 200 nM) for 6 hours until the fresh culture medium was changed. Cells treated with serum-free medium were used as a control. After 24, 48 and 72 hours, the cell viability was determined using a CCK-8 assay kit according to the manufacturer's instructions. After adding 10 mL CCK-8 to each well, followed by a 2-hour incubation, the absorbance of each sample was measured at a wavelength of 450 nm using a microplate reader (RT-2100C, China).

### Apoptosis assay

Cells were plated in 6-well culture plates (1×10^5^ cells/well) and transfected TLPs, NTLPs or Lipo2000 entrapping integrinβ6-siRNA (the final siRNA concentration was 100nM). After incubation for 48 hours, cells were harvested, washed in PBS, and resuspended in Annexin V binding buffer. Following the instructions provided by the manufacturer, Annexin V–FITC was added to the cell suspensions and the cells were incubated for 15 minutes at 4°C. Then, PI was added and incubated for 5 minutes at 4°C. The fluorescently labeled cells were tested using a flow cytometer, and the results were analyzed by Flowjo software.

### Transwell assay for cell migration and invasion

Tumor cell migration and invasion ability were analyzed in 24-well Boyden chambers with 8-μm pore size polycarbonate membranes (Costar, Acton, USA). For invasion assay, the membranes were precoated with 50 μg Matrigel (BD Biosciences, San Diego, USA) to simulate matrix barriers. Cells (5 × 10^5^/ml) were resuspended in 200 μl serum-free medium and placed in the upper chamber, and the lower compartments were filled with 600 μl medium with 10% FBS. After incubation (incubation time for migration is 10-12 hours and for invasion is 24 hours), the cells remaining on the upper surface of the membrane were removed. Then the chambers were fixed with methanol for 10 minutes and then stained with crystal violet for 20 minutes. Cells that had invaded through the membrane to the lower filter surface were counted in five random microscopic fields.

### MMP-3 and MMP-9 activity assay

The 2×10^5^ cells were cultured in a six-well cell culture plate and then transfected with β6-siRNA loaded in Lipo2000, NTLPs and TLPS for 24 hours. The levels of secreted MMP-3 and MMP-9 in the culture supernatant were collected and subjected to enzyme-linked immune sorbent assay (ELISA) following the manufacturer's guidelines (R&D). Samples were assayed in triplicate and calibrated against a standard curve.

### Tumor growth inhibition study

BALB/C female nude mice were subcutaneously implanted with HT-29 human colon cancer cells at a final concentration of 1 × 10^7^/200 ul. Mice with tumor volumes of approximately 50 mm^3^ were selected and randomly assigned to three treatment groups. The β6-siRNA encapsulated in Lipos2000, NTLPs and TLPs were gave by intratumoral or intravenous injection twice a week at a β6-siRNA dosage of 0.4 mg/kg. After 3 weeks, the mice were sacrificed and the tumors were dislodged and weighed. The tumor volume was calculated from the following formula: (W^2^ × L)/2.

### Statistical analysis

Results were presented as the mean ± standard deviation (SD) and all measurements were performed at least three independent experiments. The statistical significance was determined by Mann-Whitney test or Student's t-test. *P* <0.05 was considered statistically significant. The statistical analyses were performed using the GraphPad Prism software (GraphPad Software, Inc.).
